# Association of endometrial thickness with live birth rates among women undergoing fresh IVF, FET, and PGT cycles

**DOI:** 10.3389/fcell.2025.1530953

**Published:** 2025-03-07

**Authors:** Wenjie Huang, Juan Tang, Liuyan Wei, Liuying Nong, Ni Tang, Xiaohua Wei, Fan Zhang, Chunling Yao, Jingjing Li, Li Fan

**Affiliations:** ^1^ Department of Reproductive Medicine, Guangzhou Women and Children’s Medical center Liuzhou Hospital, Liuzhou, Guangxi, China; ^2^ Department of Reproductive Medicine, Liuzhou maternity and Child Healthcare Hospital, Liuzhou, Guangxi, China; ^3^ Guangxi Clinical Research Center for Obstetrics and Gynecology, Liuzhou, Guangxi, China; ^4^ Liuzhou Key Laboratory of Gynecologic Tumor, Liuzhou, Guangxi, China

**Keywords:** endometrial thickness, live birth rates, ART, IVF, pregnancy outcomes

## Abstract

**Background:**

Endometrial thickness (EMT) is a crucial indicator of endometrial receptivity in assisted reproductive technology (ART). However, its relationship with pregnancy outcomes remains unclear, especially across different cycle types such as fresh *in vitro* fertilization-embryo transfer (IVF-ET), frozen-thawed embryo transfer (FET), and preimplantation genetic testing for aneuploidy embryo transfer (PGT-ET). The clinical significance of EMT and its optimal range for improving ART outcomes remain subjects of debate.

**Methods:**

This retrospective cohort study analyzed data from 80,585 ART cycles conducted between July 2008 and December 2022 at a private reproductive center, including 25,683 fresh IVF-ET, 33,112 FET, and 1,071 PGT-ET cycles. EMT was measured via ultrasound on the day of HCG administration and grouped into ranges for comparison. Primary outcomes included live birth rates (LBR) and clinical pregnancy rates (CPR) across EMT ranges. Statistical analyses, including chi-square tests, receiver operating characteristic (ROC) analysis, and adjusted risk ratio (aRR) calculations, were performed to evaluate the association between EMT and pregnancy outcomes.

**Results:**

The relationship between EMT and LBR was non-linear, with no single cutoff value. LBR varied significantly across EMT ranges, peaking at approximately 12 mm in fresh IVF-ET cycles and around 10 mm in FET and PGT-ET cycles. Higher EMT was generally associated with improved LBR and CPR, but predictive power was limited (AUC: 0.56–0.60). Compared to an EMT of 10–11.9 mm, fresh IVF-ET cycles with EMT <10 mm had significantly lower LBR (aRR: 0.60–0.86), while those with EMT ≥12 mm had higher LBR (aRR: 1.12–1.17). Similar trends were observed in FET and PGT-ET cycles, although sensitivity to EMT variations was lower, particularly in PGT-ET cycles. Miscarriage rates (MR) showed no significant differences across EMT groups.

**Conclusion:**

This study demonstrates that EMT has a non-linear association with LBR and CPR across fresh IVF-ET, FET, and PGT-ET cycles, with no single cutoff value. While higher EMT generally correlates with improved outcomes, its overall predictive value for LBR is limited. The findings underscore the need for individualized evaluation of EMT based on cycle type to optimize reproductive outcomes in ART.

## Introduction

Infertility is a significant health concern affecting numerous families, and the development of ART has brought hope to many couples facing reproductive challenges. Studies indicate that maternal age and body mass index (BMI) are important negative predictors of *in vitro* fertilization (IVF) success (Vitagliano et al., 2023; Fabozzi et al., 2024), while high-quality embryos are positively correlated with the likelihood of successful conception (Gardner et al., 2000). Additionally, successful embryo implantation requires a receptive endometrium, with EMT being a primary indicator for assessing endometrial receptivity.

EMT can be easily measured via transvaginal ultrasound and has become a common indicator for evaluating endometrial status in ART. Although numerous studies have explored the relationship between EMT and pregnancy outcomes, the findings remain controversial. A meta-analysis suggested that a thinner EMT (<7 mm) negatively affects pregnancy rates, implantation rates, and live birth rates (Gao et al., 2020), while another study failed to confirm this association (Kasius et al., 2014). Moreover,the debate over whether a critical threshold of EMT impacts LBR remains unresolved (Buyalos et al., 2022). This lack of consensus may be due to the absence of a unified standard for defining EMT, variability in EMT cut-off values, sample sizes, and failure to adjust for confounding factors such as age and BMI (Mathyk et al., 2023). As result, the clinical significance of EMT on pregnancy chances after embryo transfer remains inconclusive.

Nevertheless, clinicians and couples struggling with infertility may face the dilemma of whether to proceed with a fresh IVF cycle or opt for FET, aiming to achieve a better endometrial environment under natural conditions for subsequent cycles. Therefore, the aim of the present study was to assess the impact of EMT on LBR and CPR for fresh IVF-ET cycles, FET, and PGT-ET cycles by performing.

## Materials and methods

### Study design and participants

**FIGURE 1:**
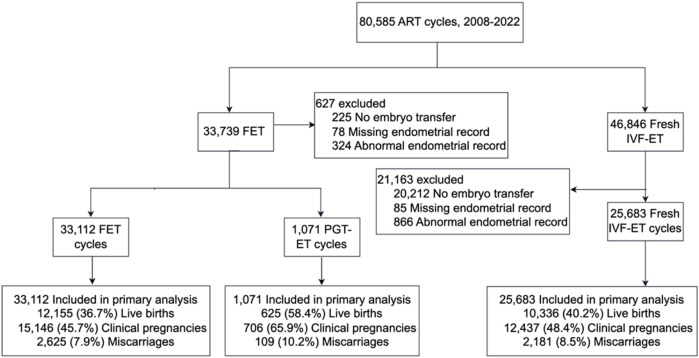
Selection, transfer cycle type, and outcomes of embryo transfer cycles evaluated through the study.

In this retrospective cohort study, we analyzed fresh ART transfer cycles, FET cycles, and PGT-ET cycles conducted at the Reproductive Medicine Center of Liuzhou Maternity and Child Health Hospital, Guangzhou Women and Children’s Medical Center, between 1 July 2008, and 31 December 2022. Ethical approval was obtained from the Ethics Committee of Guangzhou Women and Children’s Medical Center, Liuzhou Hospital (No. 2024-231). For fresh IVF-ET and FET cycles, women undergoing embryo transfer who met the study inclusion criteria were included, and cycles with inaccurate or missing EMT data were excluded (Figure 1). In PGT-ET cycles, all women undergoing single euploid blastocyst transfer were included.

Patient age was obtained from identification documents, and infertility duration was self-reported by the participants. Infertility diagnoses, ovarian stimulation protocols, and fertilization methods were recorded by physicians. Height and weight were measured on-site before treatment, and BMI was calculated (body weight in kilograms divided by the square of height in meters). Embryo-related information, including embryo grade and the number of embryos transferred, was recorded by the reproductive laboratory. Due to limitations in the earlier clinical records, embryo age was not available; however, embryo quality was assessed using grading criteria: blastocysts were classified as high quality (BB or above) and low quality (BC, CB or below), while cleavage-stage embryos were classified as grade 2 or higher for high quality. All embryos in the PGT-ET cohort were blastocysts, classified into high and low quality based on their developmental rating (De Neubourg et al., 2004). Serum hormone levels were retrieved from clinical and genetics laboratory databases.

### Controlled ovarian stimulation protocols

#### Antagonist protocol

The antagonist protocol was used for normal, high, and low responders. On days 2–3 days of the menstrual cycle, transvaginal ultrasound and blood tests were performed to confirm baseline ovarian status, after which gonadotropins (Gn) were initiated. The dose was adjusted based on age and BMI. Antagonists were added after 5–6 days of Gn or when the leading follicle reached ≥14 mm, and Gn dosage was adjusted according to monitoring results. Human chorionic gonadotropin (HCG) was administered to trigger ovulation once the dominant follicle reached the required diameter, with oocyte retrieval performed 34–36 h later. Luteal support began after oocyte retrieval, and serum HCG was measured 12–14 days after embryo transfer to confirm pregnancy. Embryo transfer occurred on day 3 or day 5 post-retrieval.

#### Agonist protocol

The agonist protocol was used for patients with conditions such as endometriosis, adenomyosis, fibroids, or repeated implantation failure. Long-acting GnRH agonists were administered on days 2–4 of the menstrual cycle, with injections given every 4 weeks for a total of 2–6 injections. Blood hormone levels were measured 14–21 days after the final injection to confirm baseline ovarian status, and Gn was started, with dosage adjusted based on age and follicle count. Ultrasound and hormone levels were regularly monitored, and HCG was used to trigger ovulation once the leading follicle reached ≥18 mm. Oocyte retrieval was performed 34–36 h later, followed by immediate luteal support, with serum HCG measured 12–14 days after embryo transfer. Pregnancy was confirmed with ultrasound, and luteal support continued until 10 weeks of gestation. Embryo transfer was performed on day 3 or day 5 post-retrieval.

#### Mild stimulation protocol

The mild stimulation protocol was employed for patients with diminished ovarian reserve or poor ovarian response in previous cycles. Baseline ultrasound was performed after menses to rule out ovarian cysts, followed by oral clomiphene or letrozole, or intramuscular HMG, starting on days 2–5 of the cycle. Follicle development was monitored after 4–5 days of treatment, and dosage was maintained if a dominant follicle was present, or increased if follicle growth was slow. When the leading follicle reached ≥16 mm and estradiol (E2) levels were appropriate, Gn was discontinued, and HCG was administered to induce ovulation. Oocyte retrieval was performed 34–36 h later, followed by luteal support and embryo transfer on day 3 or day 5. Serum HCG was measured 12–14 days after transfer to confirm pregnancy.

#### Natural cycle protocol

This protocol was used for patients with extremely poor ovarian reserve. Ultrasound monitoring began on day 8–10 of the menstrual cycle to track follicle growth. Daily monitoring was initiated once the follicle reached ≥13–14 mm, and GnRH antagonists were added if necessary. HCG or GnRH agonists were administered to trigger ovulation when the target follicle size reached ≥14–18 mm and E2 levels exceeded 150–200 pg/L. If premature ovulation occurred, the cycle was canceled. Luteal phase stimulation could be considered if applicable. Luteal support began after retrieval, and embryo transfer was performed on day 3 or day 5, with HCG testing performed 12–14 days after transfer to confirm pregnancy.

#### FET protocol

FET cycles involved either programmed cycles or natural cycles based on the physician and patient’s decision. Programmed cycles started between days 2–4 of the menstrual cycle with baseline ultrasound to rule out ovarian or uterine abnormalities, followed by oral estradiol (E2) at a starting dose of 2–4 mg, which was increased to 4–6 mg after 1 week. Vaginal estradiol could be added if needed. When EMT reached ≥8 mm and serum progesterone was <1.5 ug/L, luteal phase support with dydrogesterone 10 mg or other progestins began, and embryo transfer occurred on day 3 or day 5 post-luteal support. Natural cycles were used for patients with regular menstrual cycles and those not requiring hormone treatment. Ultrasound monitoring of follicle development began on day 10 of the cycle, with serum luteinizing hormone (LH), E2, and progesterone (P4) levels measured to confirm ovulation timing. HCG was administered to trigger ovulation when the leading follicle reached ≥16 mm, and embryo transfer occurred on day 3 or day 5 post-ovulation.

### Luteal phase support

Luteal phase support was initiated after oocyte retrieval in all controlled ovarian stimulation protocols. For fresh IVF-ET and FET cycles, dydrogesterone 10 mg/day was administered from the day of embryo transfer, with adjustments made according to individual patient needs. In some cases, additional progesterone supplementation (e.g., intramuscular or vaginal progesterone) was used. For high-risk patients, HCG was also added to prevent ovarian hyperstimulation syndrome (OHSS) if necessary. Luteal phase support continued until pregnancy was confirmed via ultrasound, approximately 33–35 days post-embryo transfer. In fresh IVF-ET cycles, serum progesterone levels were measured on the day of HCG administration to assess luteal phase adequacy. A progesterone level of ≥10 ng/mL was considered adequate for proceeding with embryo transfer, while lower levels required adjustments in luteal support. Similar protocols were followed for FET and PGT-ET cycles, with luteal support individualized based on patient characteristics.

#### Preimplantation genetic testing for aneuploidy (PGT-A)

In PGT-A cycles, controlled ovarian stimulation was performed via conventional IVF/ICSI. Oocytes were retrieved and fertilized, and embryos were cultured to the blastocyst stage (days 5–6). Trophectoderm biopsy was performed, and a few cells were analyzed for chromosomal aneuploidy using next-generation sequencing (NGS). Euploid embryos were frozen and transferred in a subsequent cycle with appropriate endometrial preparation, along with luteal support. Pregnancy outcomes were monitored through serum HCG testing and ultrasound.

#### Exposure

The exposure of interest was EMT on the day of HCG administration, with an analysis of its effect on pregnancy outcomes in fresh and frozen-thawed embryo transfer cycles. For fresh IVF-ET and FET cycles, patients were grouped based on 2-mm increments in EMT: <6 mm, 6–7.9 mm, 8–9.9 mm, 10–11.9 mm, 12–13.9 mm, 14–15.9 mm, and >16 mm. In PGT-ET cycles, due to the small sample size in the <6 mm and >16 mm groups, we combined these groups into broader categories: <8 mm, 8–9.9 mm, 10–11.9 mm, 12–13.9 mm, and >14 mm. For the analysis of LBR, the 10–11.9 mm group was selected as the reference group because it had the largest sample size and is clinically considered an ideal EMT range for implantation.

#### Outcomes

Clinical pregnancy was defined as the presence of a gestational sac on early ultrasound or the recording of live birth, ectopic pregnancy, or miscarriage. Live birth was defined as the birth of one or more living infants. Miscarriage was defined as the loss of intrauterine pregnancy before 20 weeks of gestion.

#### Statistical analyses

Continuous variables were described using the median and interquartile range (IQR), while categorical variables were expressed as frequencies and percentages. Conditional density plots (CDP) were used to visually assess the relationship between EMT and LBR for linearity. ROC curve analysis and visual inspection of CDP were used to determine whether an optimal EMT threshold for predicting live birth existed. If an optimal EMT range associated with higher LBR was observed, cross-tabulations were constructed to assess the actual distribution of observation for each 1 mm EMT increase to evaluate reliability. Mann-Whitney U and chi-square tests were used for continuous and categorical variables, respectively. Relative risks (RR) and 95% confidence intervals (CI) were calculated using log-binomial regression models. Statistical significance was set at 0.05, and all tests were two-tailed. Data were summarized using SPSS version 26.0, and R statistical language in RStudio was used for analyses.

## Results

From September 2008 to December 2022, the Reproductive Medicine Center recorded a total of 80,585 cycles, of which 46,846 (58.1%) were fresh IVF-ET cycles, and 33,739 (41.9%) were FET cycles (Figure 1). Among these cycles, 25.4% lacked transferable embryo data, and 1.7% had missing or abnormal endometrial records. Ultimately, the study included 25,683 fresh IVF-ET cycles, 33,112 FET cycles, and 1,071 PGT-ET cycles.

Demographic characteristics of the fresh IVF-ET, FET, and PGT-ET cycles are detailed in Supplementary Tables S1–S3. Increased EMT was significantly associated with younger age, lower BMI, and lower baseline FSH levels, but not with baseline E2 levels. No significant differences were observed in embryo quality or endometrial preparation methods among different thickness groups (detailed data available in Supplementary Tables).

**FIGURE 2:**
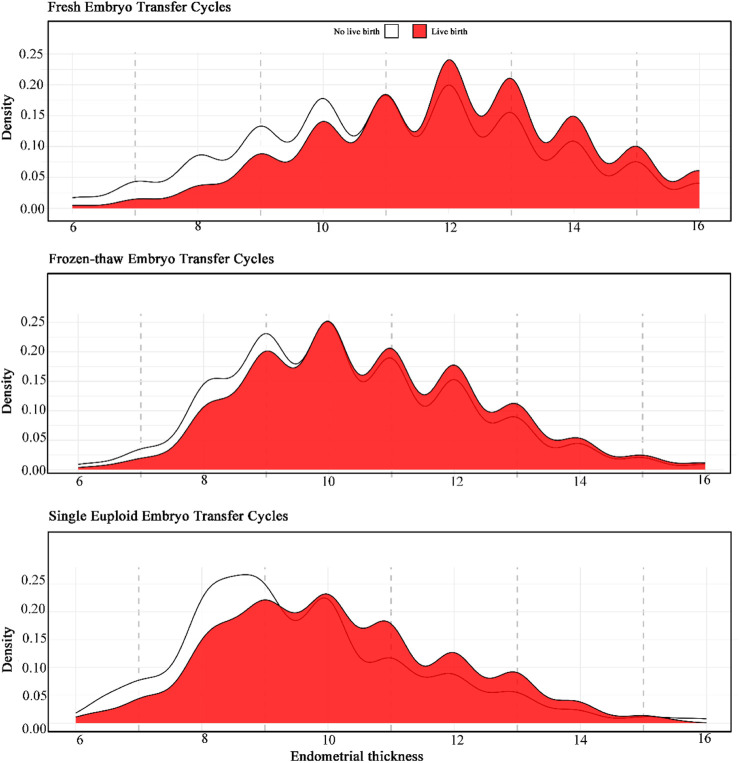
Conditional density plots of endometrial thickness in different transfer cycle types for live birth.

Our results indicate that live births occurred across different ranges of EMT in fresh IVF-ET, FET, and PGT-ET cycles. However, CDP analysis did not reveal a linear relationship between EMT and LBR, nor did it show a significant decrease in LBR below a certain threshold (Figures 2A–C). In ROC analysis, the predictive ability of EMT for live birth was low, with areas under the curve (AUC) being 0.56 for fresh IVF-ET, 0.60 for FET, and 0.59 for PGT-ET (Supplementary Figure S1), indicating limited predictive value of EMT for live births. Specifically, the peak LBR for fresh IVF-ET cycles was observed at approximately 12 mm, while for FET and PGT-ET cycles, the peak occurred at around 10 mm.

Chi-square analysis revealed statistically significant differences in LBR and pregnancy rates across different EMT ranges for fresh IVF-ET, FET, and PGT-ET cycles (p < 0.05), suggesting that variations in EMT may influence live birth and pregnancy rates in these cycles. To further analyze this, we divided EMT into high EMT (≥12 mm) and low EMT groups based on the median. Results showed that the high EMT group had a significantly higher live birth rate (48% vs 35.3%; adjusted risk ratio [aRR] 1.08 [95% CI, 1.07–1.10]) and clinical pregnancy rate (56.4% vs 43.3%; aRR 1.07 [95% CI, 1.05–1.09]) compared to the low EMT group. No significant difference in MR was found between the high EMT and low EMT groups (8.8% vs 8.3%; aRR 1.01 [95% CI, 0.97–1.05]). Similarly, in FET and PGT-ET cycles, after grouping by the median of 10 mm, the high EMT group exhibited significantly higher live birth and CPR compared to the low EMT group, showing similar trends (Supplementary Table S4).

### Fresh IVF-ET cycles

As shown in Table 1, LBR in the groups with thickness <6 mm, 6–7.9 mm, and 8–9.9 mm were significantly lower than in the 10–11.9 mm group (13.7%, 17.6%, and 28.4% vs. 38%; absolute differences of 24.3%, 20.4%, and 9.6%; unadjusted relative risks [RR] of 0.36 [95% CI, 0.23–0.52], 0.46 [95% CI, 0.41–0.53], 0.75 [95% CI, 0.71–0.79]; adjusted RR [aRR] of 0.60 [95% CI, 0.37–0.90], 0.68 [95% CI, 0.59–0.79], 0.86 [95% CI, 0.81–0.93]), controlling for potential confounding factors. In contrast, the LBR in the 12–13.9 mm, 14–15.9 mm, and ≥16 mm groups were significantly higher than in the 10–11.9 mm group (46.1%, 47.5%, and 50.2% vs 38%; absolute differences of 8.1%, 9.5%, and 12.2%; RR of 1.21 [95% CI, 1.17–1.26], 1.25 [95% CI, 1.20–1.31], 1.32 [95% CI, 1.25–1.39]; aRR of 1.12 [95% CI, 1.06–1.17], 1.12 [95% CI, 1.05–1.19], 1.17 [95% CI, 1.08–1.26]). CPR followed a similar trend, with detailed data available in Table 1. MR did not show significant differences across thickness groups.

**TABLE 1:** Clinical pregnancy, live birth, and miscarriage rate in fresh IVF-ET cycles by endometrial thickness.

Outcome	Event. No./total (%)	Absolute difference, % (95% Cl)	Relative risk (95% Cl)	
			Unadjusted	Adjusted^a^
clinical pregnancy				
10–11.9	3156 (46.2)	Reference	1 [Reference]	1 [Reference]
<6	24 (16.4)	29.8 (23.7–35.9)	0.36 (0.24–0.5)	0.54 (0.35–0.79)
6–7.9	274 (24.4)	21.8 (19–24.6)	0.53 (0.47–0.59)	0.73 (0.64–0.82)
8–9.9	1516 (37.2)	9.0 (7.1–10.9)	0.8 (0.77–0.84)	0.91 (0.85–0.97)
12–13.9	4174 (54.2)	8.0 (6.4–9.6)	1.17 (1.14–1.21)	1.1 (1.05–1.15)
14–15.9	2174 (55.8)	9.6 (7.6–11.6)	1.21 (1.16–1.26)	1.1 (1.04–1.16)
≥16	1119 (58.6)	12.4 (9.9–14.99)	1.27 (1.21–1.33)	1.15 (1.07–1.23)
Live birth				
10–11.9	2598 (38)	Reference	1 [Reference]	1 [Reference]
<6	20 (13.7)	24.3 (18.6–30)	0.36 (0.23–0.52)	0.6 (0.37–0.9)
6–7.9	198 (17.6)	20.4 (17.9–22.9)	0.46 (0.41–0.53)	0.68 (0.59–0.79)
8–9.9	1160 (28.4)	9.6 (7.8–11.4)	0.75 (0.71–0.79)	0.86 (0.81–0.93)
12–13.9	3551 (46.1)	8.1 (6.5–9.7)	1.21 (1.17–1.26)	1.12 (1.06–1.17)
14–15.9	1851 (47.5)	9.5 (7.6–11.4)	1.25 (1.2–1.31)	1.12 (1.05–1.19)
≥16	958 (50.2)	12.2 (9.7–14.7)	1.32 (1.25–1.39)	1.17 (1.08–1.26)
Miscarriage				
10–11.9	579 (8.5)	Reference	1 [Reference]	1 [Reference]
<6	4 (2.7)	5.8 (3.1–8.5)	0.32 (0.1–0.74)	0.35 (0.11–0.81)
6–7.9	85 (7.6)	0.9 (−0.8 to 2.6)	0.89 (0.71–1.11)	0.91 (0.72–1.15)
8–9.9	363 (8.9)	0.4 (−1.5 to 0.7)	1.05 (0.93–1.19)	1.06 (0.93–1.22)
12–13.9	650 (8.4)	0.1 (−0.8 to 1)	1 (0.9–1.11)	1 (0.89–1.12)
14–15.9	334 (8.6)	0.1 (−1.2 to 1)	1.01 (0.89–1.15)	1.01 (0.88–1.16)
≥16	166 (8.7)	0.2 (−1.6 to 1.2)	1.03 (0.87–1.21)	1.04 (0.87–1.23)

These analyses were tested the overall association between endometrial thickness groups and the outcomes of interest. Significant associations were observed for live birth (p < 0.001) and clinical pregnancy (p < 0.001), while the result for miscarriage (p = .21) was not significant.

^a^ Adjusted for age, BMI, AMH, infertility duration, number of embryos transfer, basal P4, Basal T, basal FSH, basal LH, basal PRL, type of infertility, embryo quality, fertilization method, infertility diagnosis, and ovarian stimulation protocol.

### FET cycles

In FET cycles, as shown in Table 2, the LBR in the <6 mm, 6–7.9 mm, and 8–9.9 mm thickness groups were significantly lower than in the 10–11.9 mm group, with diminishing differences as EMT increased (15.1%, 25.4%, and 33.4% vs. 38.1%; absolute differences of 23%, 12.7%, and 4.7%; RR of 0.40 [95% CI, 0.27–0.55], 0.67 [95% CI, 0.61–0.73], 0.88 [95% CI, 0.85–0.91]; aRR of 0.45 [95% CI, 0.30–0.64], 0.73 [95% CI, 0.66–0.81], 0.90 [95% CI, 0.86–0.94]). The LBR in the thicker groups (12–13.9 mm, 14–15.9 mm, and ≥16 mm) were significantly higher than in the 10–11.9 mm group, although the incremental improvement decreased (41.4%, 41.1%, and 43.7% vs 38.1%; absolute differences of 3.3%, 3%, and 5.6%; RR of 1.09 [95% CI, 1.05–1.13], 1.08 [95% CI, 1.01–1.15], 1.15 [95% CI, 1.02–1.28]; aRR of 1.08 [95% CI, 1.03–1.13], 1.09 [95% CI, 1.002–1.18], 1.17 [95% CI, 1.001–1.35]). The CPR in the <6 mm, 6–7.9 mm, and 8–9.9 mm thickness groups were significantly lower than in the 10–11.9 mm group (22.7%, 34.2%, and 42.3%; adjusted RR of 0.52, 0.77, and 0.91). For the 12–13.9 mm, 14–15.9 mm, and ≥16 mm thickness groups, CPR were 50.6%, 49.1%, and 49.9%, with only the 12–13.9 mm group showing a statistically significant difference (aRR of 1.06 [95% CI, 1.01–1.11]). Additionally, MR across other EMT groups did not significantly differ from the 10–11.9 mm group.

**TABLE 2:** Clinical pregnancy, live birth, and miscarriage rate in FET cycles by endometrial thickness.

Outcome	Event. No./total (%)	Absolute difference, % (95% Cl)	Relative risk (95% Cl)	
			Unadjusted	adjusted^a^
Clinical pregnancy				
10–11.9	5481 (47.4)	Reference	1 [Reference]	1 [Reference]
<6	39 (22.7)	24.7 (18.4–31)	0.48 (0.36–0.62)	0.52 (0.38–0.71)
6–7.9	507 (34.2)	13.2 (10.6–15.8)	0.72 (0.67–0.77)	0.77 (0.7–0.84)
8–9.9	4710 (42.3)	5.1 (3.8–6.4)	0.89 (0.87–0.92)	0.91 (0.87–0.94)
12–13.9	3378 (50.6)	3.2 (1.7–4.7)	1.07 (1.04–1.1)	1.06 (1.01–1.11)
14–15.9	822 (49.1)	1.7 (−0.9 to 4.3)	1.03 (0.98–1.09)	1.04 (0.97–1.12)
≥16	209 (49.9)	2.5 (−2.4 to 7.4)	1.05 (0.95–1.15)	1.06 (0.92–1.22)
Live birth				
10–11.9	4403 (38.1)	Reference	1 [Reference]	1 [Reference]
<6	26 (15.1)	23 (17.6–28.4)	0.4 (0.27–0.55)	0.45 (0.3–0.64)
6–7.9	377 (25.4)	12.7 (10.3–15.1)	0.67 (0.61–0.73)	0.73 (0.66–0.81)
8–9.9	3715 (33.4)	4.7 (3.5–5.9)	0.88 (0.85–0.91)	0.9 (0.86–0.94)
12–13.9	2763 (41.4)	3.3 (1.8–4.8)	1.09 (1.05–1.13)	1.08 (1.03–1.13)
14–15.9	688 (41.1)	3 (0.5–5.5)	1.08 (1.01–1.15)	1.09 (1.002–1.18)
≥16	183 (43.7)	5.6 (0.8–10.4)	1.15 (1.02–1.28)	1.17 (1.001–1.35)
Miscarriage				
10–11.9	1110 (9.6)	Reference	1 [Reference]	1 [Reference]
<6	13 (7.6)	2 (−2–6)	0.79 (0.44–1.27)	0.74 (0.4–1.22)
6–7.9	146 (9.8)	0.2 (−1.8 to 1.4)	1.02 (0.87–1.2)	0.99 (0.83–1.17)
8–9.9	1001 (9)	0.6 (−0.2 to 1.4)	0.94 (0.86–1.02)	0.93 (0.85–1.01)
12–13.9	625 (9.4)	0.2 (−0.7 to 1.1)	0.98 (0.89–1.07)	0.97 (0.88–1.07)
14–15.9	148 (8.8)	0.8 (−0.7 to 2.3)	0.92 (0.78–1.08)	0.91 (0.76–1.08)
≥16	166 (8.7)	2.9 (0.4–5.4)	0.7 (0.47–0.98)	0.70 (0.47–1.00)

These analyses were tested the overall association between endometrial thickness groups and the outcomes of interest. Significant associations were observed for live birth (p < 0.001) and clinical pregnancy (p < 0.001), while the result for miscarriage (p = .27) was not significant.

^a^ Adjusted for age, BMI, type of infertility, number of embryos transfer, fertilization method, and endometrial preparation.

### PGT-ET cycles

In PGT-ET cycles, as shown in Table 3, only the <8 mm thickness group had a significantly lower live birth rate compared to the 10–11.9 mm group (46% vs 63.9%; absolute difference of 17.9%; RR of 0.72 [95% CI, 0.57–0.88]; aRR of 0.72 [95% CI, 0.53–0.97]). The 8–9.9 mm group did not show a significant difference from the 10–11.9 mm group (aRR of 0.85 [95% CI, 0.70–1.02]). Furthermore, the difference in CPR between the <8 mm and 10–11.9 mm groups was not significant (aRR of 0.76 [95% CI, 0.57–1.00]). Other thickness groups (8–9.9 mm, 12–13.9 mm, and ≥14 mm) exhibited no significant differences in LBR, CPR, or MR compared to the 10–11.9 mm group.

**TABLE 3:** Clinical pregnancy, live birth, and miscarriage rate in PGT-ET cycles by endometrial thickness.

Outcome	Event. No./total (%)	Absolute difference, % (95% Cl)	Relative risk (95% Cl)	
			Unadjusted	Adjusted^a^
Clinical pregnancy				
10–11.9	230 (71.7)	Reference	1 [Reference]	1 [Reference]
<8	62 (54.9)	16.8 (6.4–27.2)	0.77 (0.63–0.91)	0.76 (0.57–1.00)
8–9.9	275 (62.1)	9.6 (2.9–16.3)	0.87 (0.78–0.96)	0.87 (0.73–1.04)
12–13.9	115 (73.2)	1.5 (−10.0 to 7.0)	1.02 (0.9–1.14)	1.02 (0.81–1.27)
≥14	24 (64.9)	6.8 (−9.3 to 22.9)	0.91 (0.68–1.12)	0.91 (0.58–1.36)
Live birth				
10–11.9	205 (63.9)	Reference	1 [Reference]	1 [Reference]
<8	52 (46)	17.9 (7.3–28.5)	0.72 (0.57–0.88)	0.72 (0.53–0.97)
8–9.9	237 (53.5)	10.4 (3.4–17.4)	0.84 (0.74–0.94)	0.85 (0.70–1.02)
12–13.9	108 (68.8)	4.9 (−13.9 to 4.1)	1.08 (0.94–1.23)	1.07 (0.84–1.35)
≥14	23 (62.2)	1.7 (−14.8 to 18.2)	0.97 (0.72–1.22)	0.97 (0.62–1.47)
Miscarriage				
10–11.9	29 (9)	Reference	1 [Reference]	1 [Reference]
<8	12 (10.6)	1.6 (−8.1 to 4.9)	1.18 (0.6–2.16)	1.11 (0.54–2.14)
8–9.9	56 (12.6)	3.6 (−8.0 to 0.8)	1.4 (0.92–2.17)	1.31 (0.84–2.08)
12–13.9	9 (5.7)	3.3 (−1.5–8.1)	0.63 (0.29–1.25)	0.66 (0.29–1.34)
≥14	3 (8.1)	0.9 (−8.4 to 10.2)	0.9 (0.22–2.37)	1.01 (0.24–2.86)

These analyses were tested the overall association between endometrial thickness groups and the outcomes of interest. Significant associations were observed for live birth (p < 0.001) and clinical pregnancy (p = 0.002), while the result for miscarriage (p = 0.14) was not significant.

^a^ Adjusted for age, type of infertility, and fertilization method.

### Subgroup analysis of first embryo transfer

To account for the potential occurrence of multiple embryo transfers within the study population, a *post hoc* subgroup analysis was conducted, including only data from the first embryo transfer in fresh IVF-ET, FET, and PGT-ET cycles. Significant differences were observed across all outcomes (LBR and CPR, both P < 0.05; MR, p > 0.05), consistent with the results of the initial analysis.

In the first fresh IVF-ET cycle, the LBR in the high EMT group was significantly higher than in the low EMT group (49.8% vs. 37.5%; absolute difference, 12.3%; aRR = 1.16 [95% CI, 1.11–1.21]). Similarly, the CPR was significantly higher in the high EMT group than in the low EMT group (58.3% vs 45.5%; absolute difference, 12.8%; aRR = 1.14 [95% CI, 1.10–1.19]). However, there was no significant difference in MR between the high and low EMT groups (8.8% vs. 8.3%; absolute difference, 0.5%; aRR = 1.06 [95% CI, 0.96–1.16]). Similarly, in the first FET and PGT-ET cycles, the LBR and CPR in the high EMT group were both significantly higher than in the low EMT group, showing a consistent trend (Supplementary Table S5).

## Discussion

In this study, we analyzed 58,795 autologous cycles and found no linear relationship or single cutoff value between EMT and LBR. However, significant associations were observed between EMT and LBR or CPR in different EMT ranges across fresh IVF-ET cycles. Specifically, the LBR was significantly lower in groups with an EMT below 10 mm compared to those with an EMT of 10–11.9 mm, while EMT ≥12 mm were associated with even higher LBRs. In contrast, EMT variations in FET and PGT-ET cycles had less impact on LBR, especially in PGT-ET cycles, where only EMT <8 mm showed significantly lower LBR compared to the 10–11.9 mm group, with no significant differences observed between other EMT ranges. This suggests that sensitivity to EMT changes differs across cycle types, indicating that optimal EMT ranges should be flexibly considered based on the cycle type in clinical practice.

A potential mechanism suggests that reduced activity of normal endometrial stem or progenitor cells, combined with insufficient response to estrogen stimulation, may lead to a thin endometrium (Gargett et al., 2016). When EMT <7 mm, low CPR might be attributed to the embryo’s proximity to spiral arteries, which could expose it to supraphysiological oxygen levels that adversely affect embryo development and implantation (Casper, 2011). Studies have shown that, compared to higher EMT, lower EMT is associated with reduced implantation and LBR across both fresh and frozen cycles (Gao et al., 2020). Our findings confirm this pattern: after adjusting for confounding factors, we observed that the high-EMT group had significantly higher CPR and LBR than the low-EMT group. However, the precise definition of a “thin endometrium” remains contentious. The threshold of EMT <7 mm for “thin endometrium” originated from a small 1990 retrospective study of 123 patients, which reported no successful pregnancies with an EMT below 6 mm (Gonen and Casper, 1990). Yet, as ART have evolved, subsequent research has shown live births with EMT as low as 4–6 mm (Sundström, 1998; Baradwan et al., 2018; Shakerian et al., 2021). In our study, fresh cycles with EMT <6 mm showed an LBR of 13.7%, while FET cycles had an LBR of 15.1%. Moreover, our study extends previous findings by demonstrating no linear relationship or single cutoff value between EMT and LBR across fresh IVF-ET, FET, and PGT-ET cycles. CDP revealed random LBR distributions across EMT ranges, and ROC analysis further supported the lack of a linear pattern or single cutoff.

A systematic review and meta-analysis by Weiss et al. concluded that EMT was not significantly associated with pregnancy rates following ovarian stimulation in couples with unexplained infertility or mild male factor infertility (Weiss et al., 2017). Additionally, a large cohort study (n = 44,477) reported that in fresh cycles, LBR decreased by 1 mm for EMT <8 mm, and in frozen cycles, LBR decreased for EMT <7 mm (Liu et al., 2018). A related large-scale study (n = 96,000) from the same database indicated that LBR improvements leveled off in fresh cycles after EMT reached 10–12 mm and in frozen cycles after 8–10 mm (Mahutte et al., 2022). These findings suggest variations in sensitivity to EMT across transfer cycle types, with differential sensitivity within the same cycle type depending on the EMT range. In our study, using the 10–11.9 mm EMT as a reference, we observed that in fresh IVF-ET cycles, groups with EMT <10 mm had significantly lower LBR and CPR, while those with EMT ≥12 mm had significantly higher rates. Similarly, in FET cycles, LBR and CPR were significantly lower in the <10 mm group compared to the 10–11.9 mm group, with diminishing differences as EMT increased, though improvements beyond 12 mm were less pronounced than in fresh IVF-ET cycles. Since MR did not significantly differ between fresh IVF-ET and FET cycles, it can be inferred that variations in LBR and CPR are likely attributed more to implantation differences rather than pregnancy loss.

Embryo aneuploidy is considered a primary reason for implantation failure in assisted reproduction cycles (Ata et al., 2021). However, most EMT studies have not accounted for embryo ploidy status, which may reduce the accuracy of estimates. Our study found that in PGT-ET cycles, while significant LBR differences existed across EMT ranges, sensitivity to EMT changes was low. Specifically, the <8 mm group had significantly lower LBR and CPR than the 10–11.9 mm group, whereas differences among groups with EMT ≥8 mm were nonsignificant. This finding is inconsistent with another study that reported higher LBRs with EMTs of 10–12 mm, though without a significant difference from thinner endometria (Ata et al., 2023). Additionally, a recent meta-analysis suggested an association between thin endometrium and increased preterm birth risk (Fang et al., 2023); however, our data in fresh IVF-ET, FET, and PGT-ET cycles did not support this finding.

### Strengths and limitations

Although the relationship between EMT and LBR did not exhibit a linear pattern or a single cutoff value, different EMT ranges still influenced LBR and CPR across specific cycle types. Our findings have clinical implications as follows: (1) LBR was observed across a wide EMT range (2.8–16 mm), suggesting that cycle cancellation due to “thin endometrium” (EMT <7 mm) should be cautiously considered; (2) sensitivity to EMT variations differs across cycle types, providing guidance for selecting fresh versus frozen cycles; (3) for patients requiring EMT interventions, the optimal EMT range should be tailored to the cycle type, with adjusted medication timing and dosage to maximize pregnancy outcomes. The large sample size of this study is a major strength, encompassing data from IVF-ET, FET, and PGT-ET cycles.

Nevertheless, limitations exist. Firstly, as a retrospective analysis, causality cannot be established, and only associations between exposure and outcomes can be inferred. Secondly, early data lacked information on embryo age, a potential confounder, which may introduce bias. Additionally, some recipients underwent multiple transfers within the study period, potentially impacting results. To control for this, we included only the first transfer per recipient in subgroup analysis, which yielded results consistent with the primary analysis.

## Conclusion

Our study demonstrated no linear relationship or single cutoff value between EMT and LBR, with EMT having limited overall predictive power for LBR. However, significant associations were observed between EMT and LBR or CPR within specific EMT ranges. Fresh IVF-ET cycle outcomes appeared more sensitive to EMT changes, whereas FET and PGT-ET cycles were less affected. The differential sensitivity across cycle types suggests that EMT should be evaluated in a type-specific context to guide clinical decision-making. Future research should investigate combined EMT and other markers to enhance predictive power for pregnancy outcomes.

## Data Availability

The raw data supporting the conclusions of this article will be made available by the authors, without undue reservation.
