# Supplementary material for: Association of endometrial thickness with live birth rates among women undergoing fresh IVF, FET, and PGT cycles
Source: Front Cell Dev Biol. 2025 Mar 7;13:1530953. doi: 10.3389/fcell.2025.1530953 (PMC11925890; doi:10.3389/fcell.2025.1530953)
Supplement: Supplementary file 1 [file DataSheet1.docx]

**Supplemental Figure 1.**

**
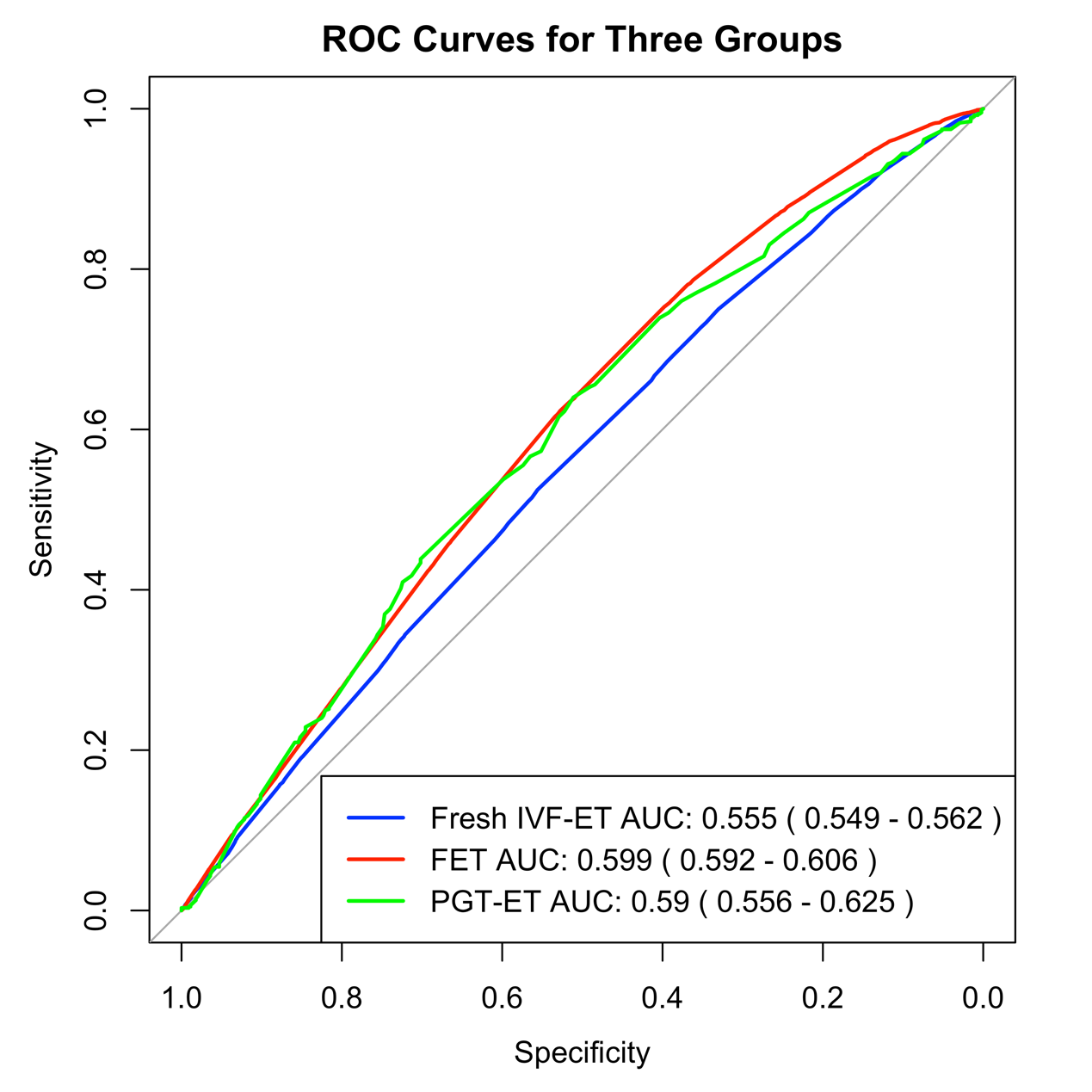
**

**Supplemental Table 1.** Baseline characteristics in Fresh IVF-ET cycle by endometrial thickness^a^

| **Characteristics median (IQR)** | < 6 mm  (146) | 6-7.9 mm  (1122) | 8-9.9 mm  (4079) | 10-11.9 mm  (6835) | 12-13.9 mm  (7698) | 14-15.9 mm  (3895) | ≥16 mm  (1908) | p |
| --- | --- | --- | --- | --- | --- | --- | --- | --- |
| **Age, y** | 37 (34-41) | 38 (34-41) | 35 (31-39) | 34 (30-37) | 33 (30-36) | 33 (29-36) | 32 (29-35) | < 0.001 |
| **BMI** | 21.42 (20.01-23.10) | 22.22 (20.21-24.115) | 21.63 (19.91-23.73) | 21.48 (19.78-23.56) | 21.36 (19.56-23.44) | 21.36 (19.72-23.44) | 21.36 (19.72-23.44) | < 0.001 |
| **AMH** | 0.5 (0.50-0.68) | 0.7 (0.65-1.08) | 0.9 (0.85-1.95) | 1.4 (1.40-2.60) | 2.3 (2.30-2.98) | 2.5 (2.50-3.12) | 2.9 (2.90-2.92) | < 0.001 |
| **Infertility duration** | 3 (2-7) | 3 (2-7) | 4 (2-7) | 4 (2-7) | 4 (2-7) | 4 (2-7) | 4 (2-7) | < 0.001 |
| **No. of embryos transferred** | 1 (1-2) | 2 (1-2) | 2 (1-2) | 2 (1-2) | 2 (1-2) | 2 (1-2) | 2 (1-2) | < 0.001 |
| **Basal P4** | 0.31 (0.20-0.52) | 0.30 (0.20-0.50) | 0.31 (0.20-0.52) | 0.34 (0.20-0.54) | 0.36 (0.22-0.57) | 0.37 (0.22-0.57) | 0.37 (0.22-0.57) | < 0.001 |
| **Basal E2** | 40.89 (27.20-57.80) | 38.46 (26.83-56.90) | 37.52 (27.34-52) | 37.50 (27.49-50.99) | 38.09 (28.12-50.66) | 37.81 (28.40-50.56) | 37.23 (28.82-50.29) | 0.096 |
| **Basal T** | 27.68 (20.00-36.62) | 26.78 (20.74-34.90) | 29.07 (21.80-39.21) | 30.01 (22.06-40.30) | 30.84 (22.40-42.14) | 30.86 (22.39-41.73) | 30.94 (22.47-41.93) | < 0.001 |
| **Basal FSH** | 7.23 (5.23-11.01) | 6.85 (5.33-9.44) | 6.4 (5.19-8.29) | 6.24 (5.11-7.79) | 6.26 (5.12-7.62) | 6.28 (5.12-7.53) | 6.29 (5.30-7.68) | 0.002 |
| **Basal LH** | 3.42 (2.22-4.95) | 3.11 (2.27-4.36) | 3.22 (2.36-4.43) | 3.32 (2.41-4.44) | 3.38 (2.51-4.56) | 3.34 (2.5-4.51) | 3.46 (2.61-4.49) | 0.003 |
| **Basal PRL** | 11.01 (8.12-14.57) | 11.58 (8.80-15.76) | 11.90 (8.86-16.47) | 12.39 (9.08-17.38) | 12.33 (9.08-17.41) | 12.33 (9.12-17.27) | 12.66 (9.24-17.52) | 0.004 |
| **Type of infertility** | |  |  |  |  |  |  | < 0.001 |
| **Primary** | 35 (24.0) | 212 (18.9) | 991 (24.3) | 2171 (31.8) | 2997 (38.9) | 1683 (43.2) | 868 (45.5) |  |
| **Secondary** | 111 (76.0) | 910 (81.1) | 3088 (75.7) | 4664 (68.2) | 4701 (61.1) | 2212 (56.8) | 1040 (54.5) |  |
| **Embryo quality** | |  |  |  |  |  |  | < 0.001 |
| **Good-quality** | 96 (65.8) | 772 (68.8) | 2939 (72.1) | 5157 (75.4) | 5890 (76.5) | 3025 (77.7) | 1418 (74.3) |  |
| **Low-quality** | 50 (34.2) | 350 (31.2) | 1140 (27.9) | 1678 (24.6) | 1808 (23.5) | 870 (22.3) | 490 (25.7) |  |
| **Fertilization method, No. (%)^b^** | |  |  |  |  |  |  | 0.01 |
| **IVF** | 121 (82.9) | 921 (82.1) | 3407 (83.5) | 5739 (84) | 6391 (83) | 3180 (81.6) | 1544 (80.9) |  |
| **ICSI** | 25 (17.1) | 201 (17.9) | 672 (16.5) | 1096 (16) | 1307 (17) | 715 (18.4) | 364 (19.1) |  |
| **Infertility diagnosis, No. (%)^c^** | |  |  |  |  |  |  | < 0.001 |
| **Male factor** | 31 (21.23) | 183 (16.31) | 622 (15.25) | 1086 (15.89) | 1424 (18.5) | 732 (18.79) | 399 (20.91) |  |
| **Tubal factor** | 82 (56.16) | 636 (56.68) | 2635 (64.6) | 4606 (67.36) | 5203 (67.59) | 2632 (67.57) | 1259 (65.99) |  |
| **Diminished ovarian reserve** | 16 (10.96) | 153 (13.64) | 267 (6.55) | 233 (3.41) | 128 (1.66) | 50 (1.28) | 13 (0.68) |  |
| **Endometriosis** | 2 (1.37) | 36 (3.21) | 116 (2.84) | 224 (3.28) | 256 (3.33) | 171 (4.39) | 88 (4.61) |  |
| **Other** | 15 (10.27) | 114 (10.16) | 439 (10.76) | 686 (10.04) | 687 (8.92) | 310 (7.96) | 149 (7.81) |  |
| **Ovarian stimulation protocol, No. (%)^d^** | | |  |  |  |  |  | < 0.001 |
| **Agonist** | 33 (22.6) | 339 (30.21) | 2263 (55.48) | 4998 (73.12) | 6475 (84.11) | 3523 (90.45) | 1806 (94.65) |  |
| **Antagonist** | 37 (25.34) | 388 (34.58) | 1213 (29.74) | 1434 (20.98) | 960 (12.47) | 289 (7.42) | 79 (4.14) |  |
| **Mild Stimulation** | 69 (47.26) | 334 (29.77) | 432 (10.59) | 269 (3.94) | 173 (2.25) | 55 (1.41) | 15 (0.79) |  |
| **Natural cycles** | 2 (1.37) | 19 (1.69) | 39 (0.96) | 27 (0.4) | 15 (0.19) | 5 (0.13) | 1 (0.05) |  |
| **Other** | 5 (3.42) | 42 (3.74) | 132 (3.24) | 107 (1.57) | 75 (0.97) | 23 (0.59) | 7 (0.37) |  |

Abbreviation: BMI (Body Mass Index) is determined by dividing a person's weight in kilograms by the square of their height in meters; IQR, interquartile range; IVF, in vitro fertilization; ICSI, intracytoplasmic sperm injection; AMH, Anti-Müllerian Hormone; P4, Progesterone; E2, Estrogen; T, Testosterone; FSH, Follicle-Stimulating Hormone; LH, Luteinizing Hormone; PRL, Prolactin.

^a^ Due to rounding, percentages may not total exactly 100%.

^b^ IVF consists of a series of advanced medical procedures that facilitate conception, while ICSI involves directly injecting a single sperm into an oocyte to promote fertilization.

^c^ Infertility diagnoses: male factor infertility, which involves sperm conception or function issues that hinder normal fertilization; tubal factor, relating to blockages or damage in the fallopian tubes; diminished ovarian reserve, indicating a lower ability of ovaries to produce eggs; Endometriosis, a condition where tissue similar to the uterine lining grows outside the uterus, causing pain, inflammation, and sometimes infertility; pelvic inflammatory disease, an infection impacting the female reproductive organs, often affecting the uterus, fallopian tubes, and ovaries, and potentially extending to the peritoneum in severe cases; and other, covering diagnoses not classified in the previous categories, such as chromosomal rearrangements or a history of medical conditions causing infertility.

^d^ The agonist protocol involves a two-step process of stimulating and then suppressing the pituitary gland, managing the environment for follicle growth; the antagonist protocol involves using medications to rapidly suppress the luteinizing hormone (LH) surge, there preventing early ovulation; mild stimulation uses smaller amounts of stimulating drugs over a shorter duration to generate fewer but potentially higher-quality eggs, thereby minimizing medication side effects and costs; Natural cycle, a fertility treatment approach that relies on the woman's natural menstrual cycle without the use of stimulating drugs, aiming to retrieve the single egg that matures naturally each month; other, including less common or individualized stimulation protocols that do not fall under the standard approaches mentioned.

**Supplemental Table 2.** Baseline characteristics in FET cycle by endometrial thickness.

| **Characteristics median (IQR)** | < 6 mm  (172) | 6-7.9 mm  (1484) | 8-9.9 mm  (11135) | 10-11.9 mm  (11556) | 12-13.9 mm  (6671) | 14-15.9 mm  (1675) | ≥16 mm  (419) | p |
| --- | --- | --- | --- | --- | --- | --- | --- | --- |
| **Age, y** | 36 (32-40) | 35 (32-39) | 34 (31-38) | 33 (30-37) | 33 (30-37) | 34 (30-37) | 33 (30-37) | < 0.001 |
| **BMI** | 21.37 (19.99-23.8) | 21.36 (19.92-23.56) | 21.3 (19.56-23.34) | 21.23 (19.56-23.31) | 21.37 (19.63-23.44) | 21.48 (19.78-23.52) | 21.64 (20.03-23.74) | < 0.001 |
| **No. of embryos transferred** | 1 (1-2) | 1 (1-2) | 1 (1-2) | 1 (1-2) | 1 (1-2) | 1 (1-2) | 1 (1-2) | 0.024 |
| **Type of infertility** | |  |  |  |  |  |  | < 0.001 |
| **Primary** | 29 (16.86) | 203 (13.68) | 2248 (20.19) | 2799 (24.22) | 1884 (28.24) | 493 (29.43) | 111 (26.49) |  |
| **Secondary** | 143 (83.14) | 1281 (86.32) | 8887 (79.81) | 8757 (75.78) | 4787 (71.76) | 1182 (70.57) | 308 (73.51) |  |
| **Embryo quality** | |  |  |  |  |  |  | 0.617 |
| **Good-quality** | 109 (63.37) | 905 (60.98) | 6610 (59.36) | 6928 (59.95) | 4009 (60.1) | 1021 (60.96) | 243 (58) |  |
| **Low-quality** | 63 (36.63) | 579 (39.02) | 4525 (40.64) | 4628 (40.05) | 2662 (39.9) | 654 (39.04) | 176 (42) |  |
| **Fertilization method, No. (%)** | |  |  |  |  |  |  | < 0.001 |
| **IVF** | 165 (95.93) | 1315 (88.61) | 9582 (86.05) | 9709 (84.02) | 5547 (83.15) | 1382 (82.51) | 330 (78.76) |  |
| **ICSI** | 7 (4.07) | 169 (11.39) | 1553 (13.95) | 1847 (15.98) | 1124 (16.85) | 293 (17.49) | 89 (21.24) |  |
| **Endometrial preparation, No. (%)** | | |  |  |  |  |  | < 0.001 |
| **Natural cycle** | 53 (30.81) | 357 (24.06) | 2919 (26.21) | 3781 (32.72) | 2260 (33.88) | 582 (34.75) | 143 (34.13) |  |
| **Programmed cycle** | 119 (69.19) | 1127 (75.94) | 8216 (73.79) | 7775 (67.28) | 4411 (66.12) | 1093 (65.25) | 276 (65.87) |  |

**Supplemental Table 3.** Baseline characteristics in PGT-ET cycle by endometrial thickness.

| **Characteristics median (IQR)** | < 8 mm  (113) | 8-9.9 mm  (443) | 10-11.9 mm  (321) | 12-13.9 mm  (157) | ≥ 14 mm  (37) | p |
| --- | --- | --- | --- | --- | --- | --- |
| **Age, y** | 34 (31-38) | 34 (30.5-37.5) | 33 (30-36) | 32 (30-36) | 33 (30-37) | 0.006 |
| **BMI** | 21.48 (19.72-23.81) | 21.83 (20.03-23.54) | 21.56 (20.2-23.31) | 21.23 (19.53-23.44) | 22.6 (21.09-24.14) | 0.153 |
| **Type of infertility** | |  |  |  |  | < 0.001 |
| **Primary** | 6 (5.31) | 62 (14) | 66 (20.56) | 38 (24.2) | 7 (18.92) |  |
| **Secondary** | 107 (94.69) | 381 (86) | 255 (79.44) | 119 (75.8) | 30 (81.08) |  |
| **Embryo quality** |  |  |  |  |  | 0.706 |
| **Good-quality** | 81 (71.68) | 309 (69.75) | 222 (69.16) | 117 (74.52) | 28 (75.68) |  |
| **Low-quality** | 32 (28.32) | 134 (30.25) | 99 (30.84) | 40 (25.48) | 9 (24.32) |  |
| **Fertilization method, No. (%)** | |  |  |  |  | 0.034 |
| **IVF** | 88 (77.88) | 351 (79.23) | 237 (73.83) | 113 (71.97) | 22 (59.46) |  |
| **ICSI** | 25 (22.12) | 92 (20.77) | 84 (26.17) | 44 (28.03) | 15 (40.54) |  |
| **Endometrial preparation, No. (%)** | |  |  |  |  | 0.076 |
| **Male factor** | 32 (28.32) | 122 (27.54) | 100 (31.15) | 62 (39.49) | 13 (35.14) |  |
| **Tubal factor** | 81 (71.68) | 321 (72.46) | 221 (68.85) | 95 (60.51) | 24 (64.86) |  |

**Supplemental table 4.** Clinical pregnancy, live birth, and miscarriage rate in fresh IVF-ET, FET and PGT-ET cycles by endometrial thickness.

| outcome | Event. No./total (%) | Relative risk (95% Cl) | |
| --- | --- | --- | --- |
| **Fresh IVF-ET cycles** | | unadjusted | adjusted^a^ |
| Clinical pregnancy | |  |  |
| ≤ 12 mm | 6790/15678 (43.3) | 1 [Reference] | 1 [Reference] |
| > 12 mm | 5647/10005 (56.4) | 1.13 (1.12-1.14) | 1.07 (1.05-1.09) |
| Live birth | |  |  |
| ≤ 12 mm | 5537/15678 (35.3) | 1 [Reference] | 1 [Reference] |
| > 12 mm | 4799/10005 (48.0) | 1.16 (1.15-1.17) | 1.08 (1.07-1.10) |
| Miscarriage | |  |  |
| ≤ 12 mm | 1296/15678 (8.3) | 1 [Reference] | 1 [Reference] |
| > 12 mm | 885/10005 (8.8) | 1.01 (0.98-1.04) | 1.01 (0.97-1.05) |
| **FET cycles** | | unadjusted | adjusted^b^ |
| Clinical pregnancy | |  |  |
| ≤ 10 mm | 7333/17427 (42.1) | 1 [Reference] | 1 [Reference] |
| > 10 mm | 7813/15685 (49.8) | 1.08 (1.07-1.09) | 1.08 (1.06-1.09) |
| Live birth | |  |  |
| ≤ 10 mm | 5777/17427 (33.1) | 1 [Reference] | 1 [Reference] |
| > 10 mm | 6378/15685 (40.7) | 1.10 (1.09-1.12) | 1.09 (1.07-1.11) |
| Miscarriage | |  |  |
| ≤ 10 mm | 1565/17427 (9.0) | 1 [Reference] | 1 [Reference] |
| > 10 mm | 1506/15685 (9.6) | 0.99 (0.96-1.03) | 1.00 (0.97-1.04) |
| **PGT-ET cycles** | | unadjusted | adjusted^c^ |
| Clinical pregnancy | |  |  |
| ≤ 10 mm | 409/664 (61.6) | 1 [Reference] | 1 [Reference] |
| > 10 mm | 297/407 (73.0) | 1.07 (1.03-1.11) | 1.08 (1.001-1.16) |
| Live birth | |  |  |
| ≤ 10 mm | 351/664 (52.9) | 1 [Reference] | 1 [Reference] |
| > 10 mm | 274/407 (67.3) | 1.11 (1.06-1.16) | 1.11 (1.03-1.21) |
| Miscarriage | |  |  |
| ≤ 10 mm | 79/664 (11.9) | 1 [Reference] | 1 [Reference] |
| > 10 mm | 30/407 (7.4) | 0.82 (0.67-0.98) | 0.83 (0.67-1.00) |

^a^ Adjusted for age, BMI, AMH, infertility duration, basal P4, Basal T, basal FSH, basal LH, basal PRL, type of infertility, embryo quality, fertilization method, infertility diagnosis, and ovarian stimulation protocol.

^b^ Adjusted for age, BMI, type of infertility, number of embryos transfer, fertilization method, and endometrial preparation.

^c^ Adjusted for age, type of infertility, and endometrial preparation.

**Supplemental table 5.** Clinical pregnancy, live birth, and miscarriage rate in first fresh IVF-ET, FET and PGT-ET cycles by endometrial thickness.

| outcome | Event. No./total (%) | Relative risk (95% Cl) | |
| --- | --- | --- | --- |
| **First Fresh IVF-ET cycles** | | unadjusted | adjusted^a^ |
| Clinical pregnancy | |  |  |
| ≤ 12 mm | 5757/12642 (45.5) | 1 [Reference] | 1 [Reference] |
| > 12 mm | 5139/8821 (58.3) | 1.28 (1.25-1.31) | 1.14 (1.10-1.19) |
| Live birth | |  |  |
| ≤ 12 mm | 4735/12642 (37.5) | 1 [Reference] | 1 [Reference] |
| > 12 mm | 4393/8821 (49.8) | 1.33 (1.29-1.37) | 1.16 (1.11-1.21) |
| Miscarriage | |  |  |
| ≤ 12 mm | 1055/12642 (8.3) | 1 [Reference] | 1 [Reference] |
| > 12 mm | 780/8821 (8.8) | 1.06 (0.97-1.16) | 1.06 (0.96-1.16) |
| **First FET cycles** | | unadjusted | adjusted^b^ |
| Clinical pregnancy | |  |  |
| ≤ 10 mm | 4353/9737 (44.7) | 1 [Reference] | 1 [Reference] |
| > 10 mm | 5020/9694 (51.8) | 1.16 (1.13-1.19) | 1.14 (1.09-1.18) |
| Live birth | |  |  |
| ≤ 10 mm | 3477/9737 (35.7) | 1 [Reference] | 1 [Reference] |
| > 10 mm | 4136/9694 (42.7) | 1.2 (1.15-1.24) | 1.17 (1.11-1.22) |
| Miscarriage | |  |  |
| ≤ 10 mm | 841/9737 (8.6) | 1 [Reference] | 1 [Reference] |
| > 10 mm | 892/9694 (9.2) | 1.07 (0.97-1.17) | 1.06 (0.97-1.17) |
| **First PGT-ET cycles** | | unadjusted | adjusted^c^ |
| Clinical pregnancy | |  |  |
| ≤ 10 mm | 326/512 (63.7) | 1 [Reference] | 1 [Reference] |
| > 10 mm | 253/345 (73.3) | 1.15 (1.05-1.26) | 1.15 (1.001-1.36) |
| Live birth | |  |  |
| ≤ 10 mm | 284/512 (55.5) | 1 [Reference] | 1 [Reference] |
| > 10 mm | 233/345 (67.5) | 1.22 (1.09-1,36) | 1,21` (1.01-1.44) |
| Miscarriage | |  |  |
| ≤ 10 mm | 56/512 (10.9) | 1 [Reference] | 1 [Reference] |
| > 10 mm | 24/345 (7.0) | 0.64 (0.40-1.00) | 0.65 (0.39-1.04) |

^a^ Adjusted for age, BMI, AMH, infertility duration, basal P4, Basal T, basal FSH, basal LH, basal PRL, type of infertility, embryo quality, fertilization method, infertility diagnosis, and ovarian stimulation protocol.

^b^ Adjusted for age, BMI, type of infertility, number of embryos transfer, fertilization method, and endometrial preparation.

^c^ Adjusted for age, type of infertility, and endometrial preparation.
